# Economies of scale in Saudi Arabia's refining sector: An application of modern econometric models

**DOI:** 10.1016/j.heliyon.2024.e30150

**Published:** 2024-04-22

**Authors:** Mohammed Al-Mahish, Fahad Alzahrani, Raga Elzaki, Mayada Ben Slama

**Affiliations:** Department of Agribusiness and Consumer Science, College of Agriculture and Food Sciences, King Faisal University, Al-Ahsa, Saudi Arabia

**Keywords:** Cobb–Douglas production function, Economies of scale, Refinery, VAR model

## Abstract

This paper aims to reveal how the refining industry's inputs in Saudi Arabia affect its output and to forecast refining industry dynamics. The variables used in this paper are the refined petroleum products representing the dependent variable, with natural gas liquids, crude oil, labor, and capital acting as explanatory variables covering the period 1990–2020. The long run cointegration of the variables was observed. An error correction model utilizing the Cobb–Douglas production function framework was performed. Furthermore, this study applied the vector autoregressive model (VAR) and its diagnosis tests, including forecast-error variance decomposition (FEVD) and impulse response functions (IRFs).

The results indicate that natural gas liquids and crude oil have a significant influence on the refining industry's output. Although capital and labor are significant determinants of output, they do not contribute significantly to output creation in the refining industry. This might be related to some parts of the capital and human resources being directed toward supporting activities, such as administration, technical support, maintenance, transportation, logistics and assigning third-party contractors to perform the main duties related to the production process. Additionally, the petroleum refining industry requires substantial capital resources for construction and maintenance. Thus, the actual measurement of capital input's influence on output was observed in the long run. The results reveal that the refining industry's variation is influenced by both its own characteristics and that natural gas liquid, crude oil, capital, and labor factors have a significant impact on the accuracy of industry forecasts.

This study concludes that Saudi Arabia's petroleum refining industry operates under decreasing returns to scale, while the shocks in the refining industry are influenced and caused by external factors.

## 1 Introduction

The manufacturing sector is considered to be one of the main drivers of economic growth [1,2]. Since the 1970s energy crisis, Saudi Arabia has embarked on an ambitious plan to reduce its dependence on the exportation of crude oil and diversify its economy. The development of the local manufacturing industry has been an essential part of this plan due to its forward and backward linkages and its primacy in investment and job creation [3]. Between 1970 and 2021, the fraction of Saudi Arabia's gross domestic product (GDP) representing the manufacturing sector increased from 5.18 % to 11.65 % [4]. In Saudi Arabia, almost all segments of the petroleum industry (the extraction of oil and natural gas, petroleum refining, and petrochemicals) are under state ownership. Moreover, the petroleum refining industry accounts for 30.15 % of the manufacturing sector.

Despite its steady growth, the manufacturing sector in Saudi Arabia remains relatively small and faces several challenges. In addition, this sector continues to depend heavily on government support from the Saudi Industrial Development Fund and is spread across roughly 30 industrial cities across the country [5]. The degree of employment generation and economic contribution performance have also been below expectations [6]. While the government is rich in hydrocarbon resources, it lacks other natural resources and has a relatively small national labor base. The shortage in the national workforce has increased Saudi Arabia's reliance on foreign workers to maintain its industrial infrastructure.

Given its outsized hydrocarbon resource endowment and the importance of quality, uniqueness, and territorial capital reputation in international competition strategies [7], the petroleum industry must be developed and made cost-effective for the manufacturing sector to play a more prominent role in Saudi Arabia's economy. To this end, and to effectively harness technological advances, it is imperative to examine the production structure of the petroleum industry in Saudi Arabia. Despite its significance, no study has been undertaken to evaluate this issue. This study attempts to fill this gap by examining the production structure of the petroleum refining industry in Saudi Arabia and providing empirical evidence on various production and efficiency parameters, such as input elasticity and economies of scale.

Thus, the primary objective of this paper is to uncover whether Saudi refineries are functioning with economies of scale or experiencing diseconomies of scale. Additionally, this paper aims to identify which production factors contribute positively to output. Conversely, this paper hopes to pinpoint inputs that do not have a positive influence on output creation, hence, helping decision-makers reform their input mix in a way that enables refinery producers (Saudi Aramco) to achieve economies of scale. This will be important for improving the efficiency of the petroleum refining industry and Saudi Arabia's economy, as petroleum refining accounts for a third of value-added manufacturing and is considered a vital link in the oil supply chain. It also aims to unveil the forecasting of refining industry dynamics to understand the current state of its situation. Thus, this investigation can provide policymakers with a clear understanding of the key factors influencing the industry's performance, such as NGLS, crude oil, real capital, and labor.

The secondary objective of this paper is to provide students and practitioners in the applied economics field with an easy, step-by-step process for estimating economies of scale using time-series data.

### 1.1 The petroleum refining industry in Saudi Arabia: an overview

Refineries are the central plants that transform crude oil into various consumable petroleum products using chemical composition and decomposition processes, including refining distills and converting crude oil. This makes it possible to extract the quantities necessary for everyday activities (heating, transport, and cooking) as well as the amounts utilized in industrial processes (petrochemicals). This range illustrates the importance of refined petroleum products in the economy.

**Fig. 1:**
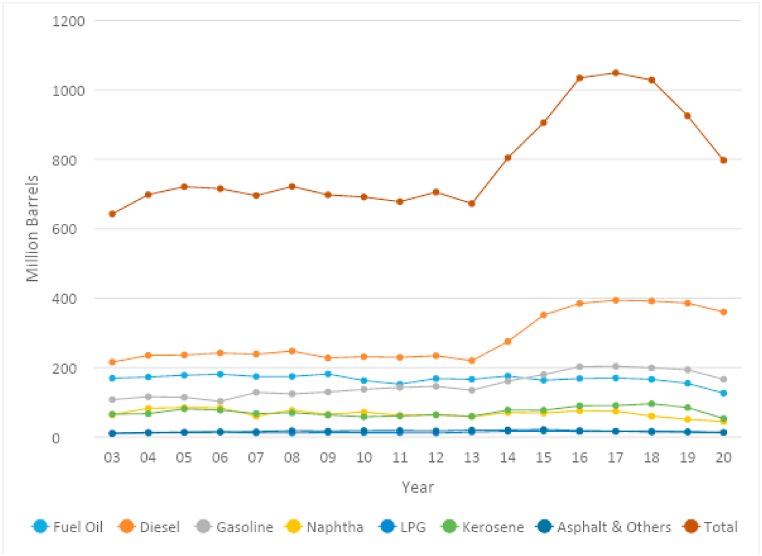
Production of refined petroleum products in Saudi Arabia from 2003 to 2020.

The petroleum refining industry in Saudi Arabia consists of 15 decision-making units, classified into three categories based on ownership: domestic refineries, domestic refinery ventures, and international refinery ventures. These refineries are entirely or partially owned by the Saudi Arabian oil company (Aramco). Aramco employs around 15,000 people in its refining, trading, and retail units. Its domestic refining capacity in 2018 was about 3 million barrels per day (MMd) [8]. During the study period, the total production of refined petroleum products in Saudi Arabia remained steady between 2003 and 2013. However, it saw a dramatic increase between 2014 and 2017. This increase in production was mainly due to the new operations started by the Yanbu Aramco Sinopec Refining Company and the Saudi Aramco Total Refining and Petrochemical Company. Production began to decrease slowly in 2018 and fell sharply in 2020 due to COVID-19, as shown in Fig. 1.

Some of the leading refined petroleum products in Saudi Arabia are fuel oil, diesel, gasoline, naphtha, liquid petroleum gas (LPG), kerosene, and asphalt. In 2020, diesel had the largest volume of refined product at 360 MMd, followed by gasoline and fuel oil at 166 MMd and 126 MMd, respectively. Most of this production is consumed domestically, either by the public sector (92.7 %) or the oil industry. Exports of refined petroleum products in 2020 amounted to about 0.37 MMd, mostly going to Western Europe (31 %), as well as Asia and the Far East (30 %) [9].

### 1.2 Literature review

In this section, we provide an overview of the literature that examines the production structure of the petroleum refining industry. We focus on studies that use parametric estimation methods, such as econometric models for production or cost functions,^1^ in their analysis. We categorize these studies based on geographical location and functional form used, specifically Cobb–Douglas (CD), constant elasticity of substitution (CES), and transcendental logarithmic (translog). Generally speaking, only a few studies have focused on the petroleum refining industry. Most of them have used translog cost functions to examine various parameters related to the production structure in the industry. However, previous empirical investigations have yielded conflicting results.

[10] examined economies of scale and economies of scope in the petroleum refining industry in the US for three refined petroleum products: motor gasoline, distillate fuels, and residual fuel. The author estimated a translog multiproduct cost function and its cost-share equations using the iterative Zellner efficient (IZEF) method and refined the operating data from 25 diversified US energy companies for the years 1981–1983. Factor prices included in the cost function were raw material (crude oil), capital, and other operating inputs. The study found that the petroleum refining industry is subject to both economies and diseconomies of scale (the long-run average cost curve exhibits an increasing, then constant, and finally decreasing returns to scale) and confirmed the presence of economies of scope in the industry, which justified the joint production of petroleum refining products [11]. also examined the economies of scale in the petroleum refining industry in the US from 1947 to 1984. They derived their estimates from cost models that function as linear and quadratic production volumes over production intervals. The authors found that larger-scale refineries (those with a production capacity of up to 500,000 barrels per day) exhibited economies of scale.

[12] investigated the existence of economies of scale across the three main government-owned branches of the Mexican petroleum industry (extraction, refining and derivatives, and basic petrochemicals). The study estimated the translog cost function and share cost equations using the IZEF method and data from 1970 to 1992. In addition to output and a time trend to represent neutral technological change, the researchers used the following factor prices in their cost function: capital, labor, and intermediate goods. They calculated the cost elasticity and corresponding economies of scale coefficient at the minimum, mean, and maximum output levels. The study found that diseconomies of scale characterized the refining industry's production levels beyond the sector's mean. They also concluded that privatizing each of the three branches of the Mexican petroleum industry would be possible without sacrificing the cost advantages gained from economies of scale.

In the Gulf Cooperation Council (GCC), another study [3] examined the different production and efficiency parameters (substitution elasticity, economies of scale, and utilization) within the petroleum refining industry in Kuwait. This was done by estimating the short- and long-run translog cost functions and sharing cost equations using the IZEF method. The study relied on annual time-series data covering the period 1976–1998. The factor prices considered in the estimations included labor, capital, and raw materials. Researchers found that the implied production structure was non-homothetic and that the pattern of scale effect was labor- and capital-saving but material-depleting. Additionally, they found that the elasticity of substitution between capital and labor was negative, implying that the two inputs are complements. Furthermore, no evidence was found for economies or diseconomies of scale in the petroleum refining industry.

Another study [13] examined nine manufacturing industries in Oman by estimating the CD production function using annual data from 1994 to 2007. The factors included in the analysis were capital and labor. The study found that the petroleum refining industry exhibited increasing returns to scale.

[14] evaluated the performance of the refining industry in Jordan by estimating two versions of the CD production functions. One version related output to both labor and capital inputs, while the other included time as an independent variable to measure disembodied technical changes. Using ordinary least squares (OLS) and data from 1967 to 1986, their results showed increasing returns to scale when estimating the production function without a time factor. However, when assessed with the time factor, the petroleum industry revealed decreasing returns to scale. According to the authors, this difference occurs because part of the technological change embodied in labor and capital inputs is not related to labor and capital technology; it was filtered out with the inclusion of time.

One study by Ref. [15] examined whether the Jordan Petroleum Refinery Company (JPRC) was a natural monopoly by evaluating the subadditivity of the cost function. Utilizing time-series data from 1961 to 2010, they estimated a system of multi-input and multi-output comprised of a translog cost function and two cost share equations using the IZEF method. Among the findings, they concluded that JPRC experienced economies of scale during the study period.

In India [16], analyzed the production behavior of the Bongaigaon refining industry by estimating CD and CES production functions using OLS and time-series data from 1990 to 2006, with capital and labor as the factors of production. The author found that, in both functional forms, the refining industry was operating under increasing returns to scale. Other researchers [17] have examined the returns to scale and elasticity of substitution of some manufacturing industries in India. They estimated a translog production function with capital and labor as inputs, using plant-level panel data for the years 1998–1999 and 2007–2008. Utilizing different estimates from each panel and fixed-effects model, their results indicated significant economies of scale for the petroleum refining industry and a positive elasticity of substitution between labor and capital. However [18], estimated a CD production function using OLS and data from 1973 to 2016, and found that the petroleum refining industry in India was operating under diseconomies of scale for the study period.

Other critical studies that have investigated various issues related to the production structure in the petroleum refining industry using different methods including [11,19,20]. A study using the VAR-BEKKG-ARCH model to derive the spillover effects of international crude oil prices on China's refined oil prices tried to forecast wholesale prices through principal component analysis. The results show that international crude oil prices have significant mean spillover and volatility spillover effects on China's refined oil wholesale prices [21]. In the same manner [22], investigated the forecasting of volatilities in crude oil and refined products, based on multivariate conditional volatility and short-memory multivariate GARCH models. They exposed that when forecasting over longer time horizons, the optimal models consisted exclusively of long-memory specifications. This finding highlights the importance of integrating long-memory characteristics for precise risk forecasts in oil markets.

According to the authors' knowledge, the production performance of the Saudi refining industry has not been empirically examined before. Therefore, the current study aims to extend the classical CD production function estimation by utilizing the error correction model framework and applying the extended method to the Saudi refining industry.

## 2 Materials and methods

The data used in this study was obtained from Saudi Arabia's Central Bank, based on data collected from the Ministry of Energy. This study utilized time-series data involving 31 observations, covering the period 1990–2020. The output variable was Saudi Arabia's aggregate refining industry output, measured in thousands of barrels. This output includes heavy fuel oil, diesel, gasoline, naphtha, LPG, kerosene, asphalt, and coke. Saudi Arabia uses crude oil and natural gas liquids (NGLs) as feedstock for producing refined products [23]. Therefore, NGLs and crude oil, measured in thousands of barrels, have been included as inputs in this study. Additionally, the quantities of labor and capital, measured in millions of Riyals, are also considered as inputs. Table 1 shows the summary statistics of the critical variables.

**Table 1:** Summary statistics of the outputs and inputs of Saudi Arabia's refining industry.

Variable	Min.	Mean	Max.	Standard Deviation
Output	517,256	695,374	1,048,887	152,439
Crude Oil	555.3	768.6	1105.8	159
NGLs	33,705	84,787	128,515	27,415
Labor	6453	12,169	22,462	4109
Capital	528	2199	3403	943

To avoid spurious regression, we conducted an augmented Dickey-Fuller (ADF) test on the log of the output and inputs of the refining industry, as the final model will be in log format. The optimal lag length was selected using the Akaike Information Criterion.

The results of the ADF test in Table 2 show that we failed to reject the null hypothesis of the unit root. Also, the null hypothesis of the unit root with a structural break in the trend and intercept was rejected in the Zivot and Andrews tests. However, when we took the first difference of the variables, we rejected the null hypotheses for both the ADF test and Zivot and Andrews test, concluding that all the variables are stationary at the first difference I(1).

**Table 2:** Unit root test results.

Variable	Variables in Level		Variables in First Difference	
Output	ADF −1.000	Zivot and Andrews −2.529	ADF −4.079***	Zivot and Andrews −7.902***
Crude oil	−1.910	−4.315	−4.272***	−9.095***
NGLs	−0.723	−4.302	−4.194***	−8.886***
Capital	−2.472	−3.409	−3.662**	−5.493**
Labor	−1.1678	−4.369	−4.475***	−5.848***

Note: *** and ** indicate significance at the 1 % and 5 % levels; respectively.

We used the Engle and Granger two-step method [24] to investigate whether the output series was cointegrated with the input series. In the first step, we estimated the following CD production function:

(1) $$\ln\;Q_{t}=\beta_{0}+\beta_{1}{\ln\;O}_{t}+\beta_{2}{\;\ln\;NGL}_{t}+\beta_{3}\;\ln\;K_{t}+\beta_{4}{\;\ln\;L}_{t}+\delta t+e_{t}$$

Where Q is the refineries' output, O is crude oil, NGL is natural gas liquid, K is real capital, L is labor, and *t* is the time-trend accounting for technological change. Augmenting model (1) with interaction terms among the independent variables means that the model becomes a multiplicative nonhomogeneous (MNH) production function, as described by Ref. [25]:

(2) $$\ln\;Q_{t}=\beta_{0}+\beta_{1}{\ln\;O}_{t}+\beta_{2}{\;\ln\;NGL}_{t}+\beta_{3}\;\ln\;K_{t}+\beta_{4}{\;\ln\;L}_{t}+\beta_{5}{\ln\;O}_{t}*{\;\ln\;NGL}_{t}+\beta_{6}{\ln\;O}_{t}*{\;\ln\;K}_{t}+\beta_{7}{\ln\;O}_{t}*{\;lnL}_{t}+\beta_{8}{\;\ln\;NGL}_{t}*\ln\;K_{t}+\beta_{9}{\;\ln\;NGL}_{t}*{\;lnL}_{t}+\beta_{10}\;\ln\;K_{t}*{\;\ln\;L}_{t}+\delta t+e_{t}$$

Furthermore, if the model (2) is extended with second-order terms, the model becomes a translog production function:

(3) $$\ln\;Q_{t}=\beta_{0}+\beta_{1}{\ln\;O}_{t}+\beta_{2}{\;\ln\;NGL}_{t}+\beta_{3}\;\ln\;K_{t}+\beta_{4}{\;\ln\;L}_{t}+\beta_{5}{\ln\;O}_{t}*{\;\ln\;NGL}_{t}+\beta_{6}{\ln\;O}_{t}*{\;\ln\;K}_{t}+\beta_{7}{\ln\;O}_{t}*{\;lnL}_{t}+\beta_{8}{\;\ln\;NGL}_{t}*\ln\;K_{t}+\beta_{9}{\;\ln\;NGL}_{t}*{\;lnL}_{t}+\beta_{10}\;\ln\;K_{t}*{\;\ln\;L}_{t}+\delta t+\beta_{11}{{\ln\;O}_{t}}^{2}+\beta_{12}{{\;\ln\;NGL}_{t}}^{2}+\beta_{13}{\ln\;K_{t}}^{2}+\beta_{14}{{\;\ln\;L}_{t}}^{2}+{\varphi t}^{2}+e_{t}$$

The second step is mainly concerned with testing the stationarity of the residuals from models (1), (2), and (3), as shown below:

(4) $$\Delta {\hat{e}}_{t}=\tau {\hat{e}}_{t-1}+\gamma_{1}\Delta {\hat{e}}_{t-1}+\gamma_{2}\Delta {\hat{e}}_{t-2}+v_{t}$$

It is important to note that Equation (4) does not contain a constant term because the mean of the regression residuals is zero [26]. The results of the cointegration test for Equation (4) are reported in Table 3.

**Table 3:** Cointegration test results.

Coefficients	CD	MNH	Translog
τ	−4.069	−3.891	−3.547
$\gamma_{1}$	1.932	1.681	1.134
$\gamma_{2}$	2.770	1.545	0.584

The test statistic for the CD model was −4.069, which is less than the critical value at the one-percent level (−3.98). Conversely, the residuals for the MNH and translog models were stationary at the 5 % level. Thus, we rejected the null hypothesis of no cointegration at the 1 % level, accepted the alternative hypothesis, and concluded that the series inputs and output of Saudi Arabia's refining industry are cointegrated in the long run. This suggests that changes in the inputs (capital, labor) will have a lasting impact on the output (refined petroleum products) of the industry over time.

Since the cointegration test indicates that all competing models are cointegrated, the next step was to select a model that best fitted the data for Saudi refineries. Table 4 shows the results of the model selection tests.

**Table 4:** Model selection test results.

Model	Chisq P-value	F-Statistics P-value
MNH vs. Translog	0.191	0.216
CD vs. MNH	0.087	0.105
CD without technological change vs. technological change	0.232	0.245

Conducting model selection tests via Chi-squared and F-tests was used to help choose between competing functional forms, such as the CD and translog models [27,28]. The results of the model selection tests, conducted via F-tests and Chi-squared tests, reject the translog model in favor of the MNH model and subsequently reject the MNH model in favor of the CD model. Thus, the preferred functional form is the CD model. Similar results were found in Ref. [17] for the petroleum refining industry in India. Additionally, the CD model with no technological change is preferred over the CD model with technological change. Thus, the model that best describes the data for Saudi refineries is the CD model without technological change. As a result, we constructed the following CD error correction model [[29], [30], [31]]:

${\Delta \ln\;Output}_{t}=\beta_{0}+\beta_{1}{\Delta \ln\;Output}_{t-1}+\beta_{2}{\Delta \ln\;Crude\;Oil}_{t}+\beta_{3}\Delta {\ln\;NGL}_{t}+\beta_{4}{\Delta \ln\;Capital}_{t}+\beta_{5}\Delta \ln\;{Labor}_{t}+\lambda {\hat{e}}_{t-1}+u_{t}$ (5) Also, this study tried to estimate the VAR model. The VAR model was considered by Ref. [32] and they confirmed its suitability for forecasting and policy simulation. However, obviously the future may be dissimilar to the past, at minimum considering the dynamics of the policy variable itself [33]. The analysis mainly focuses on the variance decomposed and impulse response functions (IRFs). We analyzed the VAR model because more benefits can be derived from the VAR analysis, which was found to be suitable for analyzing the dynamic interactions between multiple variables over time. It allowed for the possibility of all variables being endogenous, and it was able to capture the dynamic relationships between multiple variables, making them a flexible tool for analyzing complex systems. Thus can be used for forecasting, allowing us to make predictions about the future values of the selected variables in the system. Recently it has been largely applied in the analysis of energy and refining products.

The VAR model used a reduced simultaneous form as follows:

(6) $$Y_{t}=C+A_{1}y_{t-1}+\ldots +A_{\rho }y_{t-\rho }+{e\;}_{t}=C+AY_{t-1}^{t-\rho }+{e\;}_{t}$$

where Yt is the vector of endogenous variables, represented in our study as Q, O, NGLs, K, and L, which were forecasted. The only deterministic component in equation (6) was the constant term denoted by C (M × 1), a vector of the intercept. A is the matrix of coefficients for the ith lag (M × n) polynomial matrix in the backshift operator with lag length p (an estimation can be conveniently performed by equation-wise ordinary least squares (OLS) including 2 lags), and ${e\;}_{t}$ (n × 1) is the vector comprising the reduced-form residuals, which in general will have non-zero correlations.

## 3 Results and discussion

**Fig. 2:**
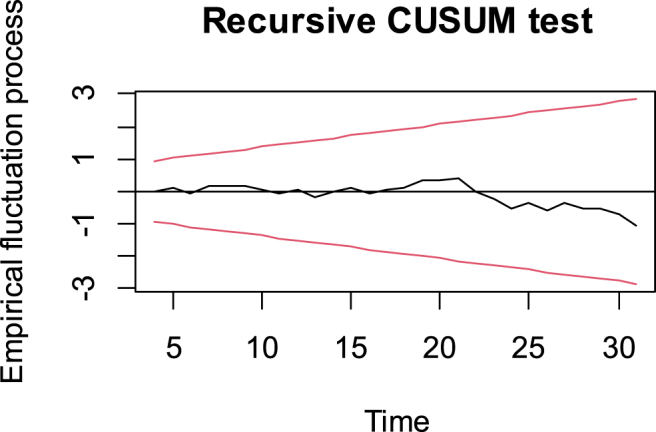
Model stability test.

To examine the validity of our model, we needed to run some diagnostic tests. Fig. 2 shows the recursive cumulative sum (CUSUM) test. The figure indicates that our model was stable. This implies that there is no evidence of significant structural changes or shifts in the relationship between the selected variables during the study period.

We also conducted a normality test to assess the normality of the model's residuals using the Shapiro-Wilk test. The results of the Shapiro-Wilk test (W = 0.96983, p-value = 0.555) indicate that we failed to reject the null hypothesis of normality. Hence, the model's residuals are normally distributed. The Durbin-Watson test shows that the model does not suffer from autocorrelation (DW-Statistics = 2.12, p-value = 0.822).

The error correction model, as shown in equation (5), was estimated with an autocorrelation heteroscedasticity robust standard error, and the estimated parameters are reported in Table 5.

**Table 5:** Estimated coefficients of the CD error correction model.

Coefficients	Model (1) Estimate	Long-Run Elasticity	Model (2) Estimate	Long-Run Elasticity
Intercept	0.012 (0.013)	–	−0.005 (0.013)	
$\beta_{1}\left({\Delta Output}_{t-1}\right)$	0.285** (0.137)	–	0.283* (0.164)	
$\beta_{2}\left(\Delta Crude\;Oil\right)$	0.411*** (0.104)	0.521*** (0.139)	0.401*** (0.110)	0.627*** (0.216)
$\beta_{3}\;\left(\Delta NGL\right)$	0.188** (0.081)	0.239** (0.095)	0.154* (0.089)	0.241* (0.138)
$\beta_{4}\;\left(\Delta Capital\right)$	−0.114*** (0.038)	−0.145* (0.080)	–	–
$\beta_{5}\;\left(\Delta Labor\right)$	−0.246*** (0.075)	−0.312*** (0.106)	–	–
${Model\;\left(2\right)\;\beta }_{4}\;\left(\Delta Capital-Labor\;Ratio\right)$	–		0.009 (0.047)	0.015 (0.079)
${Model\;\left(2\right)\;\beta }_{5}\;\left(Time\;trend\right)$	–		0.0002 (0.001)	
$\lambda {\hat{e}}_{t-1}$	−0.790*** (0.160)	–	−0.640*** (0.166)	

Note: *** and ** indicate significance at the one and five percent levels, respectively.

The CD error correction model (1) results indicate that all inputs are significant at the 5 % level or less. The error correction term is significant at the 1 % and has the expected negative sign. This term reveals that the speed of adjustment back to equilibrium in the Saudi refining industry is 79 % annually. The short-term elasticity of labor and capital is negative. This suggests that as the inputs continue to increase, the diminishing returns become more pronounced, resulting in a more substantial decline in the output per unit of additional labor or capital. While the long-term elasticity is even more negative, it means that the additional units of labor or capital contribute less to the overall refined industry's output. In model (2), capital to labor (K/L) ratio was used as an independent variable in lieu of capital and labor as separate independent variables. The coefficients of K/L in the short run and long run is positive, though insignificant.

Our finding of the negative elasticity of capital is consistent with previous research [34]; found a negative interaction between financial development and oil dependence. The negative elasticity of labor suggests that the Saudi refining industry is focused on recruiting and employing citizens, even if they do not directly contribute to refinery output. One of the primary reasons the Saudi government supports the domestic refining and petrochemicals industry is due to its significant role in job creation and unemployment reduction [35].

Additionally, Saudi Aramco has increasingly relied on outsourcing, delegating many of its duties to third-party contractors, which further justifies the negative elasticity of labor. The negative elasticity of both labor and capital confirms that not all inputs are utilized in output operations. In fact, many human and capital resources are allocated to administration, building, maintenance, supply chains, and marketing. Our results align with those of [36], who indicated that an increase in capital expenditure could result in diseconomies of scale. The misuse of resources can also occur due to poor resource management and the absence of digitization [37].

Conversely, as expected, NGLs and crude oil positively impact the refining industry's output. According to model (1), a 1 % increase in crude oil input increases the output of the refining industry by 0.41 % in the short run and 0.52 % in the long run. This positive response indicates that NGLs and crude oil play a vital role as significant energy sources and that the refining industry can contribute to the Sustainable Development Goals (SDGs) by ensuring a sufficient supply of energy products that are accessible, inexpensive, and sustainable.

Furthermore, NGLs complement crude oil by increasing the output of the refining industry by 0.19 % in the short run and 0.24 % in the long run. Our findings are consistent with those of [38], who showed that fossil fuel has a significant impact on output growth.

Additionally, the sum of input elasticities is below one, indicating that the Saudi refining industry operates under decreasing returns to scale. We also conducted an F-test to examine the null hypothesis that the Saudi refining industry operates under constant returns to scale. The results of the F-test (F-statistics = 17.6, p-value = 0.0003) show that we reject the null hypothesis of constant returns to scale. This confirms that Saudi Arabia's refining industry operates under decreasing returns to scale. At this point, Saudi Arabia's refining industry can support the SDGs (goal 7) by focusing on improving the energy efficiency of its refining processes. Furthermore, investing in renewable energy sources, such as solar or wind power, for driving the refining operations would support the achievement of clean energy objectives.

Our findings align with the conclusions from Ref. [39] which examined economies of scale in Chinese manufacturing industries and found that the petroleum refining industry in China operates in a state of diseconomy of scale. Conversely [17,40], found increasing returns to scale in the petroleum refining industries in India and Iran, respectively.

To examine the robustness of our estimated model (1) in Table 5, we conducted a robustness check by re-estimating the model with some adjustments. Table 6 shows the results of the estimated models for the sake of the robustness check. The first model is a robust regression model estimated via iterated re-weighted least squares [41,42]. The second model is the same as the original model. However, in this model, we used 51 observations from 1970 to 2020. Labor and capital observations from 1970 to 1989 were missing and were therefore recovered using multiple imputations. Nonetheless, the short-run coefficients of both models in Table 6 are identical in sign to the results of the original model in Table 5. Thus, we can confirm that the results of the original model are robust.

**Table 6:** Robustness tests.

Coefficients	Robust Regression	Using a Larger Sample Size
Intercept	0.0091 (0.0094)	0.0111 (0.0124)
${\text{Output}}_{t-1}$	–	0.3314**(0.1475)
CrudeOil	0.4917***(0.0760)	0.3950***(0.0935)
NGL	0.1391*(0.0717)	0.0051 (0.0983)
Capital	−0.0886 (0.0634)	−0.0316**(0.0149)
Labor	−0.0150 (0.0888)	−0.2574***(0.0723)
$\lambda {\hat{e}}_{t-1}$	–	−0.8345***(0.1527)

For more reliability, conclusive analysis was performed employing vector auto regressions (VAR) as a technique for policy analysis and to assess the reliability of the forecasts of the current observed data for policy formulation. The results of the VAR analysis are displayed in Appendix (2), which suggests that the coefficient for LnNGL (-2) is statistically significant at a 1 % level. This means that changes in the availability or characteristics of NGL have an effect on the production or processing of refined products. Our results were confirmed by Refs. [43,44].

VAR diagnoses to confirm the robustness of the model were also applied. We examined the VAR eigenvalue stability condition to check the stability of the VAR model. Table 7 illustrates that no root lies outside the unit circle as each modulus value in the table is lower than one. This assessment implies that the VAR model fits the stability condition. Thus, it can be deduced that the independent variables have a stable impact on the refining industry. These results can direct policymakers in formulating effective strategies and policies to optimize the utilization of these factors (O, NGL, L, and K) in the refining sector, promoting efficiency and productivity in the industry.

**Table 7:** The eigenvalue stability condition of the VAR model.

Root	Modulus
0.896388	0.896388
0.718192 − 0.461422i	0.853645
0.718192 + 0.461422i	0.853645
0.691424	0.691424
−0.583522	0.583522
0.465668 − 0.108439i	0.478127
0.465668 + 0.108439i	0.478127
−0.206834 - 0.382484i	0.434827
−0.206834 + 0.382484i	0.434827
−0.255593	0.255593

Note: No root lies outside the unit circle. VAR satisfies the stability condition. Endogenous variables: LnQ, LnO, LnNGL, LnK, and LnL, exogenous variables: C, Lag specification: 1 and 2.

In the current study, we investigated forecast-error variance decomposition (FEVD) using the Choleskey orthogonalization technique to identify the strength horizons beyond the observed time and chose 10 periods/horizons following several studies [[45], [46], [47]]. This approach estimates simultaneous shocks/innovation effects. By estimating the shocks or innovations that affect the selected variables, policymakers can evaluate the effectiveness of former policy actions which helps policymakers understand the consequences of their policy decisions and make informed modifications or improvements. In particular, it allows policymakers to make informed decisions regarding the energy infrastructure, and energy subsidies and policymakers can harmonize their policies with the SDGs and promote sustainable development. This will support SDG 9, which aims to build a resilient infrastructure, promote inclusive and sustainable industrialization, and foster innovation.

Therefore, in this study, we took 4 years to represent the short run and 10 years to represent the long run. Table 8 shows that 39.52 % of the variation in the refining industry was caused by itself, while oil, natural gas, oil, real capital, and labor caused an increasing variation in the refining industry, contributing 23.06 %, 1.71 %, 2.71 %, and 33.00 % respectively in the short run. At the same time, crude oil, natural gas and labor caused increases in the variation of forecast errors in the refining industry throughout the 10 years, ending with 28.56 %, 15.63 % and 31.42 % in the last period (10), respectively. This indicates that the refining industry is shocked by itself with smaller percentages of forecast error than the selected other variables of forecast error throughout the 10 years.

**Table 8:** Forecast-error variance decomposition for the selected variables.

Variance Decomposition of LnQ							Variance Decomposition of Ln O					
Period	S.E.	LnQ	LnO	LnNGL	LnK	LnL	S.E.	LnQ	LnO	LnNGL	LnK	LnL
1	0.048	100.000	0.000	0.000	0.000	0.000	0.094	38.139	61.861	0.000	0.000	0.000
2	0.065	82.384	11.075	1.747	0.804	3.991	0.1121	39.772	48.264	10.054	0.157	1.753
3	0.085	54.172	19.521	1.080	0.857	24.369	0.130	32.804	42.913	7.987	2.158	14.136
4	0.103	39.518	23.059	1.710	2.707	33.005	0.146	27.591	35.741	7.877	9.250	19.542
5	0.114	32.077	22.869	4.285	3.153	37.616	0.1559	24.197	32.396	8.310	12.126	22.972
6	0.122	28.428	20.327	8.632	3.249	39.365	0.163	22.089	29.552	10.051	14.084	24.222
7	0.127	26.293	19.646	12.803	3.081	38.177	0.168	20.993	28.745	11.577	14.634	24.049
8	0.133	24.427	22.024	15.279	2.829	35.442	0.1729	20.050	29.818	12.615	14.493	23.024
9	0.138	22.835	25.686	15.880	2.652	32.948	0.177	19.322	31.654	12.839	14.088	22.097
10	0.141	21.778	28.558	15.628	2.611	31.425	0.179	18.813	33.212	12.709	13.752	21.514
Variance Decomposition of LnNGL							Variance Decomposition of LnK					
Period	S.E.	LnQ	LnO	LnNGL	LnK	LnL	S.E.	LnQ	LnOL	LnNGL	LnK	LnL
1	0.100	3.929	10.107	85.965	0.000	0.0005	0.152	4.914	1.097	4.397	89.593	0.003
2	0.120	3.203	16.304	68.389	3.011	9.094	0.206	2.809	2.232	7.335	87.059	0.565
3	0.137	5.443	21.797	59.093	3.259	10.408	0.242	2.037	2.844	7.485	87.094	0.543
4	0.142	5.539	21.503	56.222	3.041	13.695	0.2668	1.733	3.102	7.597	87.030	0.538
5	0.147	5.626	20.514	53.905	2.951	17.004	0.281	1.622	2.970	7.364	87.353	0.692
6	0.151	5.412	19.510	52.327	2.829	19.923	0.2914	1.614	2.779	7.235	87.419	0.953
7	0.154	5.165	18.667	51.679	2.793	21.696	0.299	1.600	2.666	7.172	87.232	1.330
8	0.156	4.969	18.145	51.323	3.021	22.541	0.305	1.576	2.615	7.244	86.804	1.761
9	0.161	4.801	18.210	51.083	3.3533	22.553	0.309	1.541	2.567	7.426	86.311	2.155
10	0.163	4.664	18.705	50.613	3.826	22.192	0.313	1.506	2.510	7.695	85.835	2.451
Variance Decomposition of LnL												
Period	S.E.	Ln Q	LnO	LnNGL	LnK	LnL						
1	0.092	1.987	6.694	6.412	14.516	70.390						
2	0.119	2.031	6.976	6.306	13.3164	71.371						
3	0.135	1.607	5.595	14.847	11.321	66.629						
4	0.148	1.917	5.739	20.643	9.451	62.250						
5	0.160	1.958	10.143	24.547	8.121	55.230						
6	0.170	1.963	15.788	25.424	7.491	49.334						
7	0.177	1.837	20.624	24.912	7.297	45.330						
8	0.181	1.758	23.171	24.207	7.537	43.326						
9	0.183	1.774	23.934	23.7970	8.001	42.493						
10	0.184	1.840	23.842	23.574	8.563	42.181						

Note: Cholesky Ordering: LnQ, LnO, LnNGL, LnK and LnL.

The shocks of crude oil throughout the 10 years are fluctuating, while the contribution by the innovative shock of the refining industry, natural gas, labor and real capital are significantly positive and increase throughout the 10 years. The empirical evidence from Table 8 indicates that natural gas contributed larger shocks (56.22 %) in the short run and decreases in the long run (50.61 %), and that real capital is contributed to the largest by its shocks in both the short run (87.03) and long run (85.34 %). The contributions of the refining industry and real capital to labor are minimal. Also, it was noted that the shock of labor by itself estimates the largest percentage (70.39 %) of forecast error in the short run and smallest perchance (42.18 %) in the long run. This empirical evidence strongly echoes the findings of [[48], [49], [50]].

**Fig. 3:**
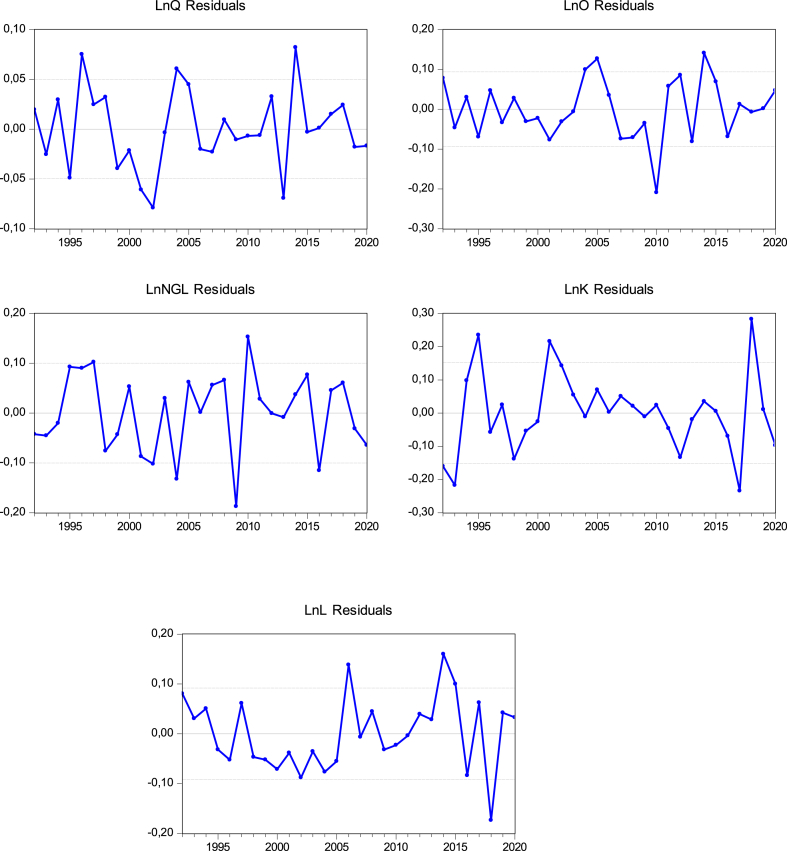
Estimated residual shocks of the selected variables. * Note: The Vertical axes represent the residuals or errors of the model generated by the VAR model, the horizontal axes represent time or observation periods.

We analyzed the residuals curve to compare the predicted and observed values of the selected variables to assess the policymakers to see whether the current implemented policies have accomplished the desired outcomes. Investigating the residuals can provide visions into the effectiveness of policy interventions and strategy involvements. In Fig. 3, the residual curves show a mixed and balanced distribution of positive and negative patterns for the selected variable, indicating that the model captures the variability in the data reasonably well. Positive residuals may show that the policy interventions regarding the refining industry have been more effective than predicted, while negative residuals may suggest that adjustments or alternative strategies are needed in this sector. Also, positive residuals in the refining industry suggest that the industry is operating more efficiently, minimizing resource utilization and waste production. It is also suggested that the refining industry may show that the energy consumption is lower than expected. Policymakers can identify best practices and encourage the adoption of technologies and processes that will reduce resource consumption, contributing to SDG 12. By improving energy efficiency, the refining sector can also contribute to SDG 7.

**Fig. 4:**
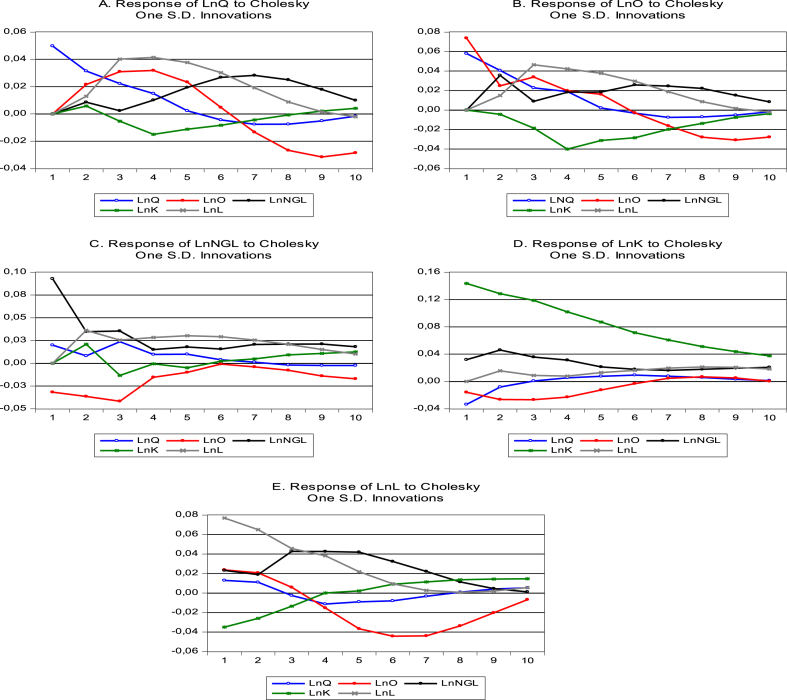
Estimated combined IRFs in the VAR model. * Note: The vertical axes represent the percentage change or deviation, the horizontal axes represent the time horizon. One S.D. Innovations refers to one standard deviation innovations or shocks in the variables.

For more validation, we analyzed the impulse response function to illustrate the response in one variable due to shocks originating from other variables. Fig. 4 plots the combined dynamic impact of one standard deviation innovation of independent variable shocks on Saudi Arabia's refining industry over a horizon of 10 years. Notably, the refining industry shows positive responses to oil, natural gas and real capital and labor during 5 horizons and shows a negative response in last horizon. This implies that these factors play a beneficial role in enhancing the industry's productivity, efficiency, profitability, and overall performance during the 5 horizons. The positive response of the refining industry to oil, natural gas, labor and real capital can support SDG 12, SDG 7 and SDG 8, respectively. In the same manner, oil shows a positive response in the short run and negative response in the long run. However, natural gas, real capital and labor show a positive response in both the short and long run.

## 4 Conclusion

Due to the importance of the petroleum refining industry to the Saudi economy and the lack of economic literature examining its productivity, this paper aimed to examine the production structure of the petroleum refining industry in Saudi Arabia. The goal was to provide empirical evidence on various production and efficiency parameters, such as input elasticity and economies of scale. It also aimed to examine forecasting for refining industry dynamics to understand the current state of the refining industry. The inputs considered in this paper included NGLs, crude oil, labor, and capital. We used annual time-series data consisting of 31 observations from 1990 to 2020.

The results show that the inputs and outputs are cointegrated in the long run, indicating a stable equilibrium or long-term connection between them. This implies that various production function specifications, such as translog, MNH, and CD, can be estimated through the error correction model framework. The model selection tests rejected the MNH and translog models in favor of the CD production function specification. Consequently, this paper developed a CD error correction model, estimated using heteroskedasticity-autocorrelation robust standard errors.

The results obtained from the analysis of long-run elasticity conclude that NGLs and crude oil have a positive and significant influence on the refining industry's output. This suggests that NGLs and crude oil variables have an important role in driving production and performance in the refining industry. This positive effect indicates a robust industrial sector which contributes to infrastructure development and technological advancements, and will achieve SDG 7.

Although capital and labor were significant determinants of refining industry output, the results indicate that they did not contribute positively to output creation. This is because human and capital resources played other supporting roles within the firms, such as administration, technical support, maintenance, transportation, and logistics. Moreover, the refining industry has realized improved output without additional capital or labor contributions, specifying improved resource utilization efficiency. Also, external factors, such as market demand and regulations, may have a stronger influence on output creation than capital and labor inputs. This might have boosted efficiency and contributed to sustainable development by optimizing resource utilization.

Finally, the null hypothesis of constant returns to scale was rejected, indicating that the Saudi refining industry has operated under decreasing returns to scale. As a result, the petroleum refining industry is a long-run investment that requires substantial capital resources to build and maintain. The precise impact of capital inputs on the petroleum refining industry, when measuring economies of scale, can only be realized in the long run. The impact of capital inputs on the petroleum refining industry and economies of scale is best observed in the long run. Capital inputs in the refining industry typically involve substantial investments in infrastructure, machinery, and technology required for planning, implementation, and integration into the production process. Additionally, the refining industry operates in a complex and dynamic market influenced by factors like crude oil prices, market demand, competition, and regulations. These factors can significantly affect long-term investment decisions and operational strategies in the industry and contribute to SDG 9 objectives. Moreover, this study reveals that the shocks in the refining industry have been caused by itself, while oil, natural gas, oil, real capital and labor have caused increasing shocks. This concludes that shocks in the refining industry are influenced or caused by external factors.

This paper recommends that policymakers in the Saudi refinery industry decrease their reliance on third-party contractors for performing the main duties related to refinery production. Policymakers should prioritize sustainability considerations in the refining industry including encouraging the adoption of environmentally friendly technologies, stimulating energy efficiency, and supporting initiatives that diminish the industry's carbon footprint, ultimately contributing to the SDG 12 targets. Also, policymakers can identify best practices and encourage the adoption of technologies and processes that will reduce resource consumption, contributing to SDG 12. By improving energy efficiency, the refining sector can also contribute to SDG 7.

Additionally, capital expenditures that do not contribute significantly to the refining production process should be reduced and allocated in line with the opportunity cost principle.

This research was limited by the unavailability of the data necessary for calculating cost or profit functions. Another limitation of this study was the lack of monthly or quarterly data, as well as a large and complete dataset of annual data, which would ensure the accuracy and reliability of the estimated model. Also, this study did not incorporate other factors such as environmental factors (emissions, fuel quality, etc.), an economic factor (GDP) and an energy transition towards being more sustainable. The suggestion of these factors and examining their connections is significant for the refining industry, for policymakers/decisions, and for investors who are making decisions and developing strategies and plans for achieving the sustainability development goals.

We recommend that future research employ cost functions to estimate economies of scope in the Saudi petroleum refining industry and confirm the expected savings from the joint production of refinery products.

## Data availability

The dataset used in this study is available in Appendix 1. In addition, the data is available through the following link: https://www.sama.gov.sa/en-US/EconomicReports/Pages/YearlyStatistics.aspx.

## CRediT authorship contribution statement

**Mohammed Al-Mahish:** Writing – review & editing, Writing – original draft, Visualization, Supervision, Software, Project administration, Methodology, Funding acquisition, Formal analysis, Data curation. **Fahad Alzahrani:** Writing – review & editing, Writing – original draft, Project administration, Investigation, Conceptualization. **Raga Elzaki:** Writing – review & editing, Visualization, Validation, Methodology, Formal analysis. **Mayada Ben Slama:** Writing – original draft, Conceptualization.

## Declaration of competing interest

The authors declare the following financial interests/personal relationships which may be considered as potential competing interests: Mohammed Al-Mahish reports article publishing charges was provided by King Faisal University.

## Figures and Tables

**Table:** 

Year	Production (in thousand barrels)	Capital (in million Riyals)	Number of workers	NGL consumption (in thousand barrels)	Oil (in thousand barrels)
1990	561,236	528	6453	33,705	698.08
1991	517,256	569	7009	35,019	580.89
1992	541,529	590	7185	45,263	640.42
1993	548,809	587	7168	47,655	640.48
1994	551,631	790	7751	53,083	662.63
1995	529,211	1231	7758	68,853	632.41
1996	600,601	1326	7837	72,781	729.44
1997	578,288	1547	9796	73,494	666.95
1998	584,099	1542	10,373	71,944	689.79
1999	581,529	1536	10,716	69,223	673.42
2000	602,154	1545	10,629	75,284	680.22
2001	597,455	2014	10,744	66,676	676.36
2002	582,879	2362	10,562	72,278	660.09
2003	642,623	2429	10,628	73,972	688.89
2004	698,114	2431	10,658	65,603	769.53
2005	720,640	2645	10,672	80,563	782.7
2006	715,427	2771	12,150	77,022	795.18
2007	695,369	3025	12,387	90,915	676.61
2008	721,365	3137	12,873	96,318	693.92
2009	697,511	3204	13,054	85,455	699.61
2010	691,109	3204	13,366	116,594	555.34
2011	677,701	3212	13,444	113,492	763.93
2012	705,313	2746	15,519	113,357	789.62
2013	672,232	2732	17,288	100,799	754.3
2014	803,844	2733	20,919	110,540	934.13
2015	905,427	2885	22,462	121,611	1105.77
2016	1,034,262	2751	18,853	106,022	1096.84
2017	1,048,887	2181	18,537	121,481	1091.85
2018	1,028,298	3217	13,401	127,314	1074.52
2019	924,942	3403	13,230	128,515	1011.06
2020	796,862	3293	13,808	113,572	913.09

**Table:** 

Independent Variables	Dependent Variables				
	LnQ	LnO	LnNGL	LnK	LnL
LnQ (-1)	0.325042	0.153498	0.752047	0.566641	0.016972
	[ 1.04376]	[ 0.26125]	[ 1.19939]	[ 0.59702]	[ 0.02957]
LnQ (-2)	−0.034014	−0.320518	0.448497	−0.033082	−0.342459
	[-0.16589]	[-0.82853]	[ 1.08636]	[-0.05294]	[-0.90626]
LnO (−1)	0.265297	0.420833	−0.516895	−0.167263	0.007275
	[ 1.54713]	[ 1.30076]	[-1.49709]	[-0.32004]	[ 0.02302]
LnO (−2)	0.038715	0.195824	−0.399375	−0.050268	0.009360
	[ 0.27182]	[ 0.72873]	[-1.39265]	[-0.11580]	[ 0.03566]
LnNGL (-1)	0.022284	0.329185	0.167396	0.121572	−0.016320
	[ 0.21776]	[ 1.70496]	[ 0.81242]	[ 0.38979]	[-0.08654]
LnNGL (-2)	−0.241445	−0.257283	0.404115	−0.026577	0.305835
	[-2.13047]***	[-1.20326]	[ 1.77098]	[-0.07695]	[ 1.46429]
LnK (-1)	0.081478	0.015975	0.259342	0.944212	0.025041
	[ 0.92819]	[ 0.09645]	[ 1.46731]	[ 3.52926]	[ 0.15479]
LnK (-2)	−0.028001	−0.106641	−0.365806	−0.116305	0.007675
	[-0.29277]	[-0.59099]	[-1.89960]***	[-0.39900]	[ 0.04354]
LnL (-1)	0.168365	0.193022	0.469987	0.201596	0.845267
	[ 1.24669]	[ 0.75754]	[ 1.72840]	[ 0.48978]	[ 3.39612]***
LnL (-2)	0.246829	0.175644	−0.225049	−0.365917	−0.123841
	[ 1.65019]	[ 0.62239]	[-0.74725]	[-0.80267]	[-0.44924]
Constant	5.701012	1.201538	−6.667566	−3.914073	3.378806
	[ 3.01603]***	[ 0.33691]	[-1.75188]	[-0.67941]	[ 0.96991]
R-squared	0.960244	0.845199	0.925337	0.941649	0.942370
Adj. R-squared	0.938158	0.759198	0.883858	0.909232	0.910354
Sum sq. resids	0.044556	0.158606	0.180635	0.413878	0.151335
S.E. equation	0.049753	0.093869	0.100176	0.151635	0.091692
F-statistic	43.47648	9.827816	22.30835	29.04800	29.43386
Log-likelihood	52.78619	34.37585	32.49013	20.46826	35.05632
Akaike AIC	−2.881806	−1.612128	−1.482078	−0.652983	−1.659056
Schwarz SC	−2.363177	−1.093498	−0.963448	−0.134354	−1.140427
Mean dependent	13.44736	6.637651	11.34792	7.647667	9.391068
S.D. dependent	0.200066	0.191291	0.293947	0.503308	0.306244

Note: t-statistics in [ ]. Source: Authors' calculation.

## Footnotes

1 N.N. Dalei, J.M. Joshi, Estimating technical efficiency of petroleum refineries using DEA and tobit model: An India perspective, Comput. Chem. Eng. 142 (2020). https://doi.org/10.1016/j.compchemeng.2020.107047. See Dalei and Joshi (2020) for a review of studies that use Data Envelopment Analysis (DEA), a non-parametric method, for analysing petroleum refineries. We do not cover those here since DEA requires data on individual decision-making units (DMU), which we do not have access to.
